# Editorial Chit Chat

**Published:** 1871-04

**Authors:** 


### EDITORIAL CHIT CHAT.

QUEER.—A young lady friend of ours, residing in Indianapolis, is about to take to her Coffin, on her wedding day. We are inclined to think however, that he will make an acceptable brother-in-law.

PRACTICING UNDER DIFFICULTIES.—Mormon physicians are required, under a penalty of $1,000, to explain the nature of every dose of medicine administered by them, and the effect they expect it to have. Brigham must have a better educated class of physicians in his domain than are generally found among us, else would they soon lose the confidence of their patients by constantly misjudging the action of their remedies.

CHLORAL PREPARATIONS.—We never advertise nor recommend patent medicines of any kind, as our readers are aware. In giving Paine Brother's Chloral preparations a place in our columns, we do so knowing the proprietors to be honorable gentlemen, and their Chloral preparations to be all they claim for them.

The Chloral Soother is a convenient form in which to administer this excellent anodine—it is agreeable to the taste, which is a great advantage in its administration.

A NEW LODGE.—H. W. McIntyre, long and favorably known to the Masonic Fraternity of this city and State, has been appointed to a lucrative position with an Alaska fur company, called the Alaska Commercial Co., and has gone to that distant country to enter upon his duties. We expect soon to hear of a Masonic Lodge organized at his station, for Mac couldn't be comfortable, even wrapped in furs, without being near a lodge. He will have one, depend upon it, if he is compelled to take a seal for a secretary, and a polar bear for a tiler.

BENEVOLENCE OF GERMAN SURGEONS.—The German surgeons in the Prussian army, had a most beautiful system of pinning to the breast of every wounded Frenchman, a diagnosis or accurate description of his injury, so that when the French ambulances called for the exchanged wounded, the patient was not subjected to the pain of a second examination of his wound, but was treated as recommended by the German surgeon's card upon his breast. This is a humane and unique system, worthy the noble and generous corps of German surgeons.

ONLY THINK OF IT!—Chemists tell us that when we pour milk into a cup of tea, the albumen of the milk and the tannin of the tea instantly unite and form leather, or minute flakes of the very same compound which is produced in the texture of tanned hides. It is also estimated that in the course of a year an ordinary tea drinker will imbibe enough leather to make a pair of shoes. Therefore, persons who do not desire to convert their stomachs into first-class tanneries, had better discontinue the use of milk in their tea.

MEDICAL LIBRARIES.—There are compartively few large medical libraries in the world, for the reason that medical works are more costly than any other class of books. The best works on medicine and surgery—in the various specialties of the science, cost from five to fifty, eighty, and even one hundred dollars a volume. The largeest medical libraries in this country are owned by Drs. Purple, Stephen Smith, Carroll and W. A. Hammond. They have from two to three thousand volumes in their respective libraries. Their books will average at least eight dollars apiece, making a library of three thousand volumes a very costly luxury.

HIGH—VERY.—We desire no better proof of the fallacy of spiritualism, than the fact that the spirit of a deceased lady allowed a minister, in a neighboring village, to write the following obituary of her, without her returning and knocking his high-fa-lutin head off from him:—"Death, with fleshless knuckles, rapped at the door of Mrs.——'s soul, and obedient to the inexorable call, the spirit of that loved woman floated up to its Creator, leaving her husband, children and friends, to mourn over the mortal casket." What a confounded pity, death didn't try his knuckles on the parson's skull and knock him out of time, too!

NEW DOCTORS.—The different medical colleges of the country, will graduate fifteen hundred doctors of medicine this year. What will become of them? Whither will they go to find employment in their chosen profession? While there is room for skillful and successful physicians, there is none whatever, for the ordinary sort. And as not one physician in five hundred, rises to any eminence in the profession, it strikes us, that a great many of the new doctors, just graduated, will have to seek employment in an entirely different field.

Entirely too many of our young men choose medicine for a profession, thinking it an easy and profitable mode of gaining a livelihood. They soon find their mistake however, after entering upon their duties as a physician. To be successful, they must first possess *tact* for the business, then, having graduated as doctors of medicine, they have but entered upon their student life, which never ceases, until the day of their deaths. Their toil is incessant, their charities manifold, and their pay slender. To become a successful physician is a life-long labor, and we imagine but very few of the young gentlemen just graduated, have either the tact or ability to rise above mediocrity.

AN ELEGANT BED.—The one manufactured by the "Metallic Union Spring Bed Co.," at Poughkeepsie, N. Y. We are using one of their beds, and pronounce it to be the very best we have ever seen. It is entirely unlike the metallic beds we have been in the habit of seeing in this section—nothing like them. It is entirely metallic, perfectly level, uniformly elastic, as much so at the edges as in the centre; can be raised and curved in every possible direction, and it is just the thing for a sick bed, as it can be changed into so many shapes, is perfectly clean, and as soft and elastic as a feather bed. Address the Company as above and secure their price list.

SOMETHING NEEDED.—The people have become distrustful of the many Life Insurance Companies that have sprung into existence. Extravagance in the management of companies, the lavish expenditures upon agents and their offices, reckless and wild speculations with the funds of the companies, by their officers, have lead to much loss to policy holders. Our state has given us a wholesome and much needed law, regulating the management of Life Insurance Companies, and securing the people against loss. An Insurance Superintendent is appointed by the state, whose duty it is to require bonds and mortgages, not less than $25,000, from each company, to be deposited with him, as a security to policy holders. A new company, just started in New York, has gone even farther than this—it deposits U. S. Bonds, or other reliable securities, with the superintendent, to the amount of every policy held against it, so securing the holder against any possibility of loss. The policy is endorsed by the state superintendent, assuring the holder that securities to the amount of the policy, has been deposited with him, as security, for the holder. That is the sort of a company to insure with. It is called the "Government Security Life Insurance Co.," and is ably represented by Solomon Bowles, Esq., in this city.

A MODEL MANAGER.—One of the ladies of Mr. J. F. Sherry's excellent theatrical troupe, informs us that during the six years which she has traveled with the company, she has never known Mr. Sherry to fail to pay his people promptly, that he has never been compelled to disband his company for want of funds, or patronage, that he has always paid all claims against him upon demand, that he never drinks intoxicating liquors, never swears, and is universally beloved and respected by his troupe. That is what not one manager in a thousand can have said of him. We know Mr. Sherry to be a courteous Sir Knight, and a gentleman.

A NEW SWINDLE.—M. House, a divorce lawyer of New York, whose card has appeared in most of the newspapers of the country, in which he claimed to procure "absolute divorces legally in any State, without publicity," &c., has finally come to grief and his mode of doing business exposed. He has been in the habit of having a partner appear before the judges of the various courts, who makes oath that his name is the same of the real party who applied for the relief, and making his case strong enough, the decree is issued and afterwards handed to the real applicant, for a sum varying from one hundred to one thousand dollars. When the party applying is a woman, she is also represented by a female partner, who does the necessary swearing, and with whom the divorce lawyer shares the spoils. It appears that large numbers have been so divorced, and those who have re-married find themselves defendants in suits for bigamy. Be careful who you employ to secure a divorce for you. Quack lawyers are as tricky as quack doctors.

PERSONAL.—We are indebted to Mr. Frances Collingwood, one of the Engineers of the great bridge, now under course of construction between New York and Brooklyn, for a very pleasant visit. He gave us a very interesting description of the compressed air, in the CAISSON, upon the healths of the workmen. The great weight of the atmosphere upon the "drums" of the ear, produces considerable pain, until the air reaches the middle ear, through the *eustachian tubes*, and gives support to the membrane. The air also has a very exhilarating effect, and quickens the circulation, so that the pulse will run from 70 up to 150. Several cases of paralysis have occurred among the workmen employed within the caisson, under the influence of the compressed air. It becomes very difficult to extinguish a candle, from the increased supply of oxygen to the flame. An Irishman takes no pleasure from smoking his pipe, as his tobacco burns something like gunpowder, leaving no time for the enjoyment of the weed. Altogether, the caisson must be a curious place to visit.

MALPRACTICE.—In the great majority of instances of suits for malpractice, against surgeons of undoubted ability, the object of the prosecutor is to black-mail the surgeon, thinking that he would rather pay a handsome fee and have the matter hushed up, than to have his reputation suffer through a suit for malpractice. We are glad to know that our courts have taken this matter into consideration, and in a recent trial of Dr. Sayre, of New York, and Dr. Reese, of Philadelphia, the jurys not only acquitted the physicians named, but assessed damages upon the prosecutors, for needlessly subjecting the physicians to the expense of defending themselves in a suit for malpractice, when no cause appeared for so doing.

Judge Thayer's charge to the jury in the case of Dr. Reese, was as follows:

"The implied contract of a physician or surgeon who attends a patient is, not that he will entirely effect a cure, but that he will *use all known and reasonable means* to accomplish that object, and that he will attend his patient carefully and dilligently. His relation to his patient implies that he possesses, and will employ in the treatment of the case, such *reasonable* skill and diligence as are *ordinarily* exercised in his profession by thoroughly educated surgeons or physicians. No presumption of the absence of proper skill and attention arises from the mere fact that the patient does not recover or that a complete cure is not effected. God forbid that the law should apply any rule so rigorous and unjust as that, to the relations and responsibilities arising out of this noble and humane profession. On the part of the patient, it is his duty to conform to the necessary prescriptions and treatment, if they be such as a surgeon of ordinary skill and care would adopt or sanction, and if he will not, or, under the pressure of pain cannot, the surgeon or physician is not responsible for injury resulting therefrom."

The jury, without leaving the room, returned a verdict for the doctor, and assessed the costs upon the plaintiff. In Dr. Sayres' case, who was charged with producing lameness in opening an abscess of the hip, the jury acquitted him of the charge and assessed $1,500 upon the prosecutor to defray the expenses of the Doctor in defending his suit. This is as it should be.

### PERIODICALS AND BOOKS.

LYCEUM BANNER, published by E. H. Kimball, at 137½ Madison Street, Chicago, Ill., at one dollar a year, is an excellent little monthly for children.

THE HERALD OF HEALTH.—This is a very popular health magazine, published monthly, by Wood & Holbrook, 13 & 15 Laight Street, New York, at two dollars per annum. We will furnish it, with the BISTOURY, at the same price.

THE AMERICAN STOCK JOURNAL.—A very useful journal for stock breeders and farmers. Contains a vast amount of useful information. Is published by N. P. Boyer & Co., at Parkersburg, Pa., for one dollar a year. We will also furnish this journal with the BISTOURY, at the same price—one dollar.

AN EXCELLANT MEDICAL JOURNAL.—"The Medical Times," a semi-monthly journal of medical and surgical science, published by J. B. Lippencott & Co., of Phil., at four dollars a year. It is the best printed, and best edited medical journal in America. Send for a specimen copy.

"GOOD HEALTH."—A first class health journal for the people. Ably edited by the best medical writers of Boston—issued monthly, at two dollars a year, by Alexander Moore, 11 Broomfield Street, Boston. By sending two dollars to THE BISTOURY, we will furnish you our own journal, and "Good Health," for one year.

THE GAS CONSUMERS' GUIDE.—A popular Hand Book of instruction, on the proper management and economical use of gas, with a full description of gas meters, and directions for ascertaining the consumption, by meter, &c. Illustrated, 12 mo. cloth, $1.00, paper, 75 cts. Alexander Moore, Pub., Boston.

LITTLE CORPORAL.—This beautiful little magazine for children, continues to be as lively, chatty, and entertaining as ever. Is carefully edited, and is the most entertaining journal for the little folks that we know of. Price $1.50 a year. Published at Chicago, by John E. Miller. We will furnish it with the BISTOURY, at the same price.

A NEW BOOK BY DR. STORER.—We are in receipt of a work on the "Causation, Course and Treatment of Reflex Insanity in Women," by Horatio Robinson Storer, M. D., L. L. B., of Boston. It is received too late for a review in this number of The Bistoury. The reputation of the author is a sufficient guarantee to the profession, that the work is a valuable one.

A MODEL JOURNAL.—A model of typographical neatness, is Geo. P. Rowell & Co's Newspaper Reporter. It is a weekly magazine, printed upon beautifully tinted, heavy paper, and gives much useful information to Newspaper men. Messrs. Rowell & Co., have done much for the publishers of America, by furnishing their columns with advertisements from reliable parties, and doing the tedious work of collecting their dues for them, without expense or trouble to the publishers.

SATIN IN SOCIETY.—A real good, sensible book. Written in plain, comprehensive and chaste language, and should be a present from the father to every son, of fourteen years of age, in America. Fathers are too careless in instructing their sons upon the necessity for avoiding certain evil practices, which are by far too common among the youth of our land—leading them to early vices and shattered health. This book gives the desired advice and warning. *Every boy needs it.* Let him have it! C. F. Vent, Publisher, Cincinnatti, O. Price, $1.50

A MUCH NEEDED WORK.—Dr. Geo. H. Napheys has written another timely book, one that has been long needed by the youth of our land. It contains information that should be in the possession of every young man. Thousands have been ruined mentally and physically for the want of just such knowledge as this book affords. It is entitled "Councels on the Nature and Hygeine of the Masculine Function." Price $2,00, It will be sent by mail, post-paid, on receipt of price, by Hall Brothers, of this city.

